# Age-Associated Changes in Recombinant H5 Highly Pathogenic and Low Pathogenic Avian Influenza Hemagglutinin Tissue Binding in Domestic Poultry Species

**DOI:** 10.3390/ani11082223

**Published:** 2021-07-28

**Authors:** Carmen Jerry, David E. Stallknecht, Christina Leyson, Roy Berghaus, Brian Jordan, Mary Pantin-Jackwood, Monique S. França

**Affiliations:** 1 California Animal Health and Food Safety Laboratory System, University of California, Davis, 1550 N. Soderquist Road, Turlock, CA 95380, USA; 2 Southeastern Cooperative Wildlife Disease Study, 589 D.W. Brooks Drive, Athens, GA 30602, USA; dstall@uga.edu; 3 Southeast Poultry Research Laboratory, U.S. National Poultry Research Center, Agricultural Research Service, U.S. Department of Agriculture, 934 College Station Road, Athens, GA 30602, USA; christina.leyson@usda.gov (C.L.); mary.pantin-jackwood@usda.gov (M.P.-J.); 4 Veterinary Medical Center, Department of Population Health, 2200 College Station Road, Athens, GA 30602, USA; berghaus@uga.edu; 5 Poultry Diagnostic and Research Center, University of Georgia Athens, 953 College Station Road, Athens, GA 30602, USA; brian89@uga.edu (B.J.); mfranca@uga.edu (M.S.F.)

**Keywords:** age, influenza, highly pathogenic, low pathogenic, ducks, turkeys, chicken, recombinant, hemagglutinin, tropism

## Abstract

**Simple Summary:**

We evaluated differences in avian influenza H5 Hemagglutinin (HA) tissue binding across age groups using recombinant H5 HA (rHA) proteins. Gene sequences from low pathogenic (LPAIV) (A/mallard/MN/410/2000(H5N2) and an a high pathogenic (HPAIV) (A/Northern pintail/Washington/40964/2014(H5N2)) influenza A virus (IAV) were used to generate the rHA proteins. Respiratory and intestinal tracts from chickens, ducks (Mallard, Pekin, Muscovy), and turkeys of different age groups were used to detect rHA binding with protein histochemistry, which was quantified as the median area of binding (MAB) for statistical analysis. Turkeys had significant differences in the HPAIV rHA binding in the respiratory tract, with younger turkeys having higher levels of binding in the lung compared to the older group. In the intestinal tract, younger turkeys had higher levels of binding compared to the older birds. Using LPAIV, only turkeys had overall significant differences in the respiratory tract MAB, with the older birds having higher levels of binding compared to the younger group. We saw no age-related differences in the overall intestinal tract rHA binding. Age-related differences in rHA binding of the LPAIV and HPAIV rHA demonstrated in this study may partially, but not completely, explain differences in host susceptibility to infection observed during avian influenza outbreaks and in experimental infection studies.

**Abstract:**

The 2014 outbreak of clade 2.3.4.4A highly pathogenic avian influenza (HPAI) led to the culling of millions of commercial chickens and turkeys and death of various wild bird species. In this outbreak, older chickens and turkeys were commonly infected, and succumbed to clinical disease compared to younger aged birds such chicken broilers. Some experimental studies using waterfowl species have shown age-related differences in susceptibility to clinical disease with HPAI viruses. Here, we evaluate differences in H5 Hemagglutinin (HA) tissue binding across age groups, using recombinant H5 HA (rHA) proteins generated using gene sequences from low pathogenic (A/mallard/MN/410/2000(H5N2 (LPAIV)) and a HPAIV (A/Northern pintail/Washington/40964/2014(H5N2)) influenza A virus (IAV). Respiratory and intestinal tracts from chickens, ducks (Mallard, Pekin, Muscovy) and turkeys of different age groups were used to detect rHA binding with protein histochemistry, which was quantified as the median area of binding (MAB) used for statistical analysis. There were species and tissue specific differences in the rHA binding among the age groups; however, turkeys had significant differences in the HPAIV rHA binding in the respiratory tract, with younger turkeys having higher levels of binding in the lung compared to the older group. In addition, in the intestinal tract, younger turkeys had higher levels of binding compared to the older birds. Using LPAIV, similar species and tissues, specific differences were seen among the age groups; however, only turkeys had overall significant differences in the respiratory tract MAB, with the older birds having higher levels of binding compared to the younger group. No age-related differences were seen in the overall intestinal tract rHA binding. Age-related differences in rHA binding of the LPAIV and HPAIV demonstrated in this study may partially, but not completely, explain differences in host susceptibility to infection observed during avian influenza outbreaks and in experimental infection studies.

## 1. Introduction

The hemagglutinin (HA) surface glycoprotein is a major determinant of influenza A virus (IAV) entry to host cells and is the predominant surface glycoprotein [1,2,3]. The HA exists as a trimer with a receptor binding pocket on each subunit [1,4,5]. Binding of influenza viruses to cells is mediated by the attachment of the receptor binding site on viral HA to terminal sialic acid on glycoconjugates on the surface of cells [1,6,7,8]. There is fine coordination between the HA and the neuraminidase (NA), which acts as a sialidase to cleave sialic acid and to facilitate virus entry through receptor mediated endocytosis [9].

The sialic acid repertoire for several poultry, domestic and wild birds has been established in the literature. França et al., described the sialic acid repertoire of 11 wild birds [10]. The respiratory and intestinal tract of waterfowl had predominantly α2, 3-linked sialic acid receptor. However, ducks also had α2, 6-linked sialic acid in the lower intestine. In terrestrial birds, both α2, 3 and had α2, 6-linked sialic acid were present in the respiratory tract and lower intestine [10]. Another study by Kimble et al. examined the sialic acid receptor distribution in gallinaceous species, and noted that geese and ducks had significant expression of α2, 3-linked sialic acid receptor in the respiratory tract and small amounts of α2, 6-linked sialic acid in the colon [11]. Turkeys expressed both α2, 3 and α2, 6 sialic acid in the respiratory tract and intestinal tract, and additionally, there was increased expression of α2, 6 in older birds [11]. Kuchipudi et al. also evaluated differences in sialic acid receptors between chickens and ducks. In chickens, the dominant receptor type was as α2, 6-linked sialic acid, whereas in ducks, the dominant receptor type was α2, 3 [12]. Age-related differences in sialic acid receptor expression in respiratory and intestinal tissue was described by Pillai et al. using chickens, turkeys, and ducks of different age groups and slight variations in the expression level of sialic acid receptors were noted among the age groups [13].

Numerous experimental studies report age-related differences in susceptibility to infection with IAVs in waterfowl and poultry species [14,15,16,17,18,19]. Löndt et al., used a clade 2.2 H5N1 virus to evaluate age-related pathogenicity in 8- and 12-week-old Pekin ducks (*Anas platyrhynchos domesticus*) and found that 8-week-old birds developed neurologic disease, and died by the fourth day post infection, while the older birds developed mild clinical signs with no mortality [17]. Pantin-Jackwood et al. described an age-related susceptibility to H5N1 HPAIV experimentally, where 2-week-old Pekin ducks developed more severe neurologic signs and higher mortality compared to 5-week-old ducks used in the same study [20]. Other experimental infections of Pekin ducks with H5N8 HPAIV clade 2.3.4.4 viruses show that ducks become infected at doses of 10^4^ EID_50_ and 10^6^ EID_50_, and developed pyrexia and neurologic signs with no mortality [21]. The ducks in the aforementioned study shed virus mostly through the oropharyngeal route, for up to 7 days post infection, and less frequently and at lower titers via the cloacal route [21,22].

Using juvenile (4- to 8-week-old) and adult Ruddy ducks (*Oxyura jamaicensis*) and juvenile Lesser scaups (*Aythya affinis*), Spackman et al., examined the pathogenesis of infection with clade 2.3.4.4 H5 viruses and showed that juvenile Ruddy ducks had higher mortality at high doses of inoculation compared to the adults, suggesting that age may be a contributing factor to susceptibility [23]. Additionally, experimentally infected 16-week-old, broad breasted white turkeys (*Meleagris gallopavo*) survived longer compared to 6-week-old birds, which had a shorter mean death time [24]. These age-related differences in susceptibility to infection may partially be explained by alterations in the expression of sialic acid receptors as birds become older [11,12,15,25,26]. For instance, in turkeys, there is a positive correlation between increasing age and the expression of α2, 6 sialic acid receptors in the intestine [11,27]. Therefore, distribution of sialic acid receptors alone, do not completely account for differences in viral tropism seen in vivo. Other confounding factors such as host immunity, glycosylation of the hemagglutinin (HA), fine co-ordination of the neuraminidase with the HA, host adaptability of the HA, and route of infection should be considered in interpretation of results from HA binding studies.

Age-related susceptibility of broiler and broiler breeder chickens (*Gallus gallus domesticus*) to clade 2.3.4.4, A/turkey/Minnesota/12582/2015 (H5N2) was investigated by Bertran et al. [28]. In this study, no significant differences in the mean death times, and virus titers shed orally were observed among the age groups. It was therefore concluded that this virus was not adapted to broiler chickens and some level of host genetic resistance and increased biosecurity could have accounted for the broilers being unaffected in the 2014 HPAIV outbreaks [28].

Age-related susceptibility to infection with LPAIV was described among gallinaceous birds. Brugh et al. found differences in morbidity rates in leghorn chickens inoculated with H5N2, H4N8, and H3N2 low pathogenic influenza viruses concluding that older birds had mildly higher morbidity compared to the younger chickens [29]. In another study, Costa et al. evaluated the effect of age on viral shedding of LPAIV in mallards (*Anas platyrhynchos*) showing that 4-week-old birds consistently shed the virus via the cloacal route compared to the other age groups [30]. Additionally, França et al. demonstrated by lectin histochemistry the distribution of α2, 3 sialic acid receptors in uninfected and infected (H3N8, H5N2 LPAIV) 2-week to 4-month-old mallards. High levels of *Maackia amurensis* 2 lectin (MAA2) bound to the respiratory and intestinal tract of infected and uninfected birds indicating high levels of α2, 3-linked sialic acid across age groups. Only the nasal turbinate had an age dependent increase in MAA2 staining.

Considering the collective findings in the literature, we investigated whether an age-related difference in the hemagglutinin tissue binding could explain some of the findings in the field and experimentally. Recombinant hemagglutinin glycoproteins (rHA) were generated using the segment 4, from the genome of a HPAIV (A/Northern pintail/Washington/40964/2014(H5N2)) and a LPAIV (A/mallard/MN/410/2000(H5N2)). The HPAIV belonged to the clade 2.3.4.4A, isolated during the 2014 outbreak in the United States and a LPAIV isolated from a mallard in 2000 [21,31].

## 2. Materials and Methods

### 2.1. Generation of rHA for HPAIV and LPAIV

For the generation of the rHA, we used a high-pathogenicity and low-pathogenicity H5 influenza virus. HA gene sequences from Influenza A virus A/Northern pintail/Washington/40964/2014(H5N2) (GenBank accession number KP307976, highly pathogenic) and Influenza A virus A/mallard/MN/410/2000(H5N2) (GenBank accession number: EF607875, low pathogenic) were obtained from NCBI GenBank using methods previously described [32,33]. Codon optimization was performed on the amino acids at positions 18-564 and a GCN4 trimerization domain was appended to the HA gene sequence at the carboxy terminal end with a StrepTag [32,34].

We cloned the HA constructs into the mammalian expression vector pEXPR-IBA42 using restriction enzyme digestion and ligation [32]. The vector and insert were then ligated by combining digested fragments in a 1:3 ratio (vector: insert) with T4 DNA ligase (Fermentas/Thermo Scientific, Vilnius, Lithuania) [32]. The mixture was incubated at 4 °C overnight and then introduced to chemically competent *E. coli* cells (JM109, Promega, Madison, WI, USA), using heat shock method per the manufacturer’s protocol [32]. We plated the cell mixture to Luria Bertani (LB) agar plates with 100 µg/mL Carbenicillin. An overnight culture of LB broth and 100 µg/mL Carbenicillin, was placed in shaking incubator and used for plasmid purification using Zymo Maxiprep kit^®^ (Irvine, CA, USA) according to the manufacturer’s directions [32,33].

We filter sterilized the purified plasmids with a 0.22 µm syringe filter and Sanger sequencing of the plasmids was done by the Georgia Genomics facility (Athens, GA, USA) [32,33]. 

The expression vectors with the HA domains were transiently transfected into human embryo kidney cells (HEK 293T cells) [32,33]. At 5–6 days post transfection, the cell media was harvested and then used for protein purification using StrepTactin^®^, (IBA life sciences, Goettingen, Germany) affinity chromatography [32]. A Nanodrop^®^ (Wilmington, DE, USA) was used to quantify proteins produced by absorbance at 280 nm [32,33]. Confirmation of the expression of the recombinant proteins was done by SDS-PAGE and Western blot using StrepTactin-HRP^®^ (IBA life sciences, Goettingen, Germany) on cell culture media as well as purified proteins [25,32,33,34].

### 2.2. Protein Histochemistry

The respiratory, intestinal tract tissue and cloacal bursa from freshly dead mallards, Muscovy ducks, Pekin ducks, turkeys, and chicken egg layers were selected and fixed in 10% neutral buffered formalin for less than 24 h [32]. For each age group, three birds per species were sampled. Tissues were then processed routinely for histopathology and embedded in paraffin. Tissue microarrays were constructed using an Arraymold^®^ (IHC World Life science products and services, Woodstock, MD, USA) using manufacturer’s protocol [32]. 

Briefly, tissue sections were deparaffinized, placed in a steamer for 45 min in pH 6 Citrate buffer for antigen retrieval and endogenous peroxidases were blocked using Bloxall© (Burlingame, CA, USA) for 10 min at room temperature, and then the tissue was incubated in 10% normal goat serum for 30 min at room temperature [32]. The protein histochemistry was then performed as described by Leyson et al. and Jerry et al. [32,33]. 

### 2.3. Quantification of rHA Binding in Tissues

Recombinant HA binding was detected as dark brown staining of the cells in different organs with protein histochemistry [32]. For each bird, images of the respiratory, intestinal, and cloacal tissue were obtained with an Olympus BX41 light microscope (Melville NY, USA) at 20× magnification with Olympus cellSens Standard software (Olympus Lifescience, Waltham, MA, USA). We then used Fiji software (https://imagej.net/software/fiji/, accessed on 2 October 2019) to convert the regions of rHA binding to a numerical value, indicated as the area of binding in µm^2^. The median area of binding (MAB) was then determined from the three birds in each age group and used for statistical analyses using Graph Pad Prism 9.0. (GraphPad Software, San Diego, CA, USA) The cumulative MAB was determined by adding the MAB scores from trachea and lung to obtain the overall respiratory MAB and the upper and lower intestine for the overall intestinal MAB. For descriptive purposes, we used a scale for rHA MAB binding follows: 0–10 µm^2^: no binding, >10–500 µm^2^: mild, >500–1000 µm^2^ moderate, >1000–1500 µm^2^: strong, >1500 µm^2^: very strong [32].

### 2.4. Statistical Analysis

For both the HPAIV H5 rHA and the LPAIV H5 rHA, the Kruskal–Wallis test was used to determine a statistically significant difference in the medians for each tissue, among different age groups belonging to the same species [32]. The Dunn’s post-test was then used to determine if there was a pairwise statistically significant difference among bird and age groups in each species [32]. Where two age groups were evaluated, the Mann–Whitney U test was used for pairwise comparisons. A *p* value of ≤ 0.05 was considered statistically significant. 

## 3. Results

### 3.1. Highly Pathogenic Avian Influenza H5 rHA

**Figure 1:**
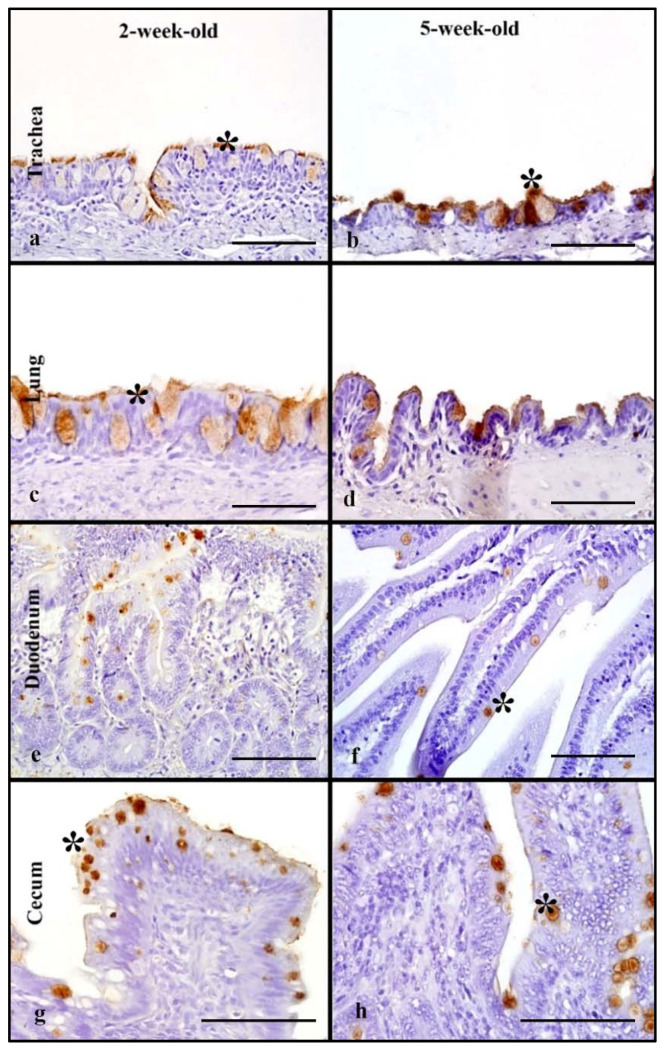
HPAIV H5 rHA binding in Mallard ducks of different age groups, visible as dark brown staining using 3, 3-diaminobenzimidine (DAB) HRP via protein histochemistry, denoted by asterisk (*). In the trachea (**a**,**b**), strong levels of rHA binding is seen in 2-week-old and 5-week-old Mallards. Note higher levels of HA binding in the lung of the 2-week-old (**c**) compared to the 5-week-old. In the upper intestine, slightly stronger levels of rHA binding is seen in the 2-week-old (**e**) compared to the 5-week-old. Note similar, very strong levels of rHA binding in the cecum of both 2-week-old and 5-week-old birds. Scale bar is 50 µm.

**Figure 2:**
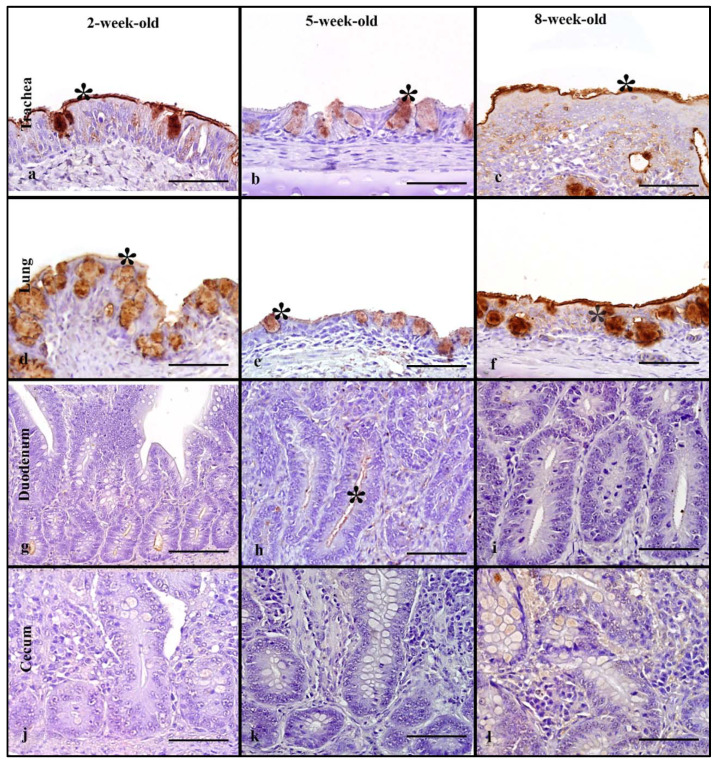
HPAIV H5 rHA binding in Muscovy ducks of different age groups, visible as dark brown staining using 3,3-diaminobenzimidine (DAB) HRP via protein histochemistry denoted by asterisk (*). Strong and very strong levels of rHA bindngi is seen in trachea of 2- and 8-week-old Muscovy ducks (**a**,**b**). Note very strong levels of binding in the lung of 2- and 8-week-old Muscovy ducks (**d**,**f**). Minimal to mild rHA binding is seen in the intestinal tract of Muscovy ducks (**g**–**l**). Scale bar is 50 µm.

**Figure 3:**
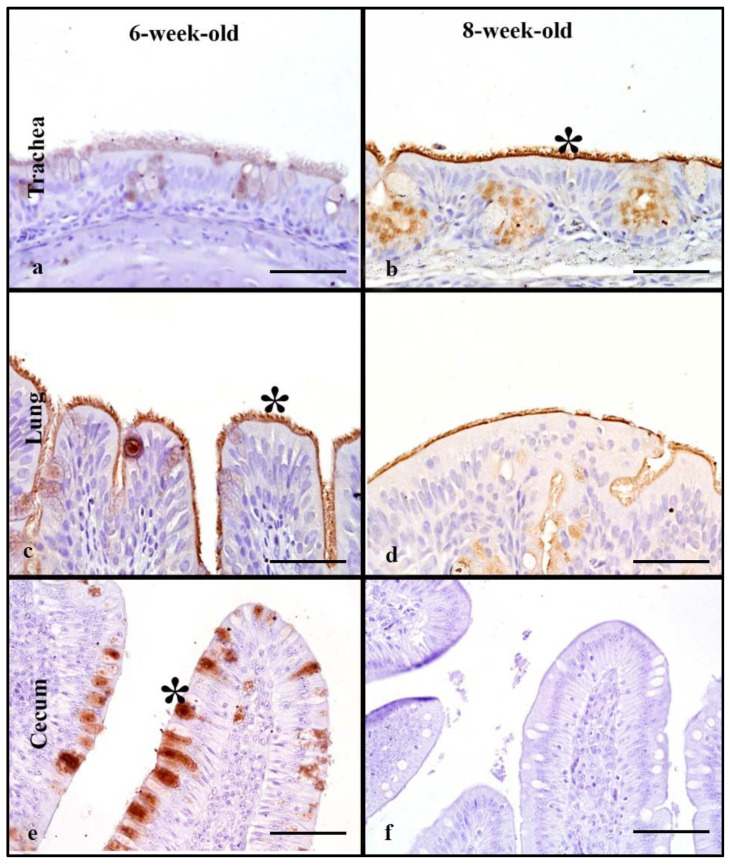
HPAIV H5 rHA binding in turkeys of different age groups, denoted by dark brown staining using DAB- HRP via protein histochemistry, denoted by asterisk (*). Very strong rHA binding to the trachea in the 8-week-old turkeys (**b**) compared to milder binding in the 6-week-old birds (**a**). Very strong rHA binding to the lung and cecum of 6-week-old turkeys is also evident (**c**,**e**). Scale bar is 50 µm.

**Figure 4:**
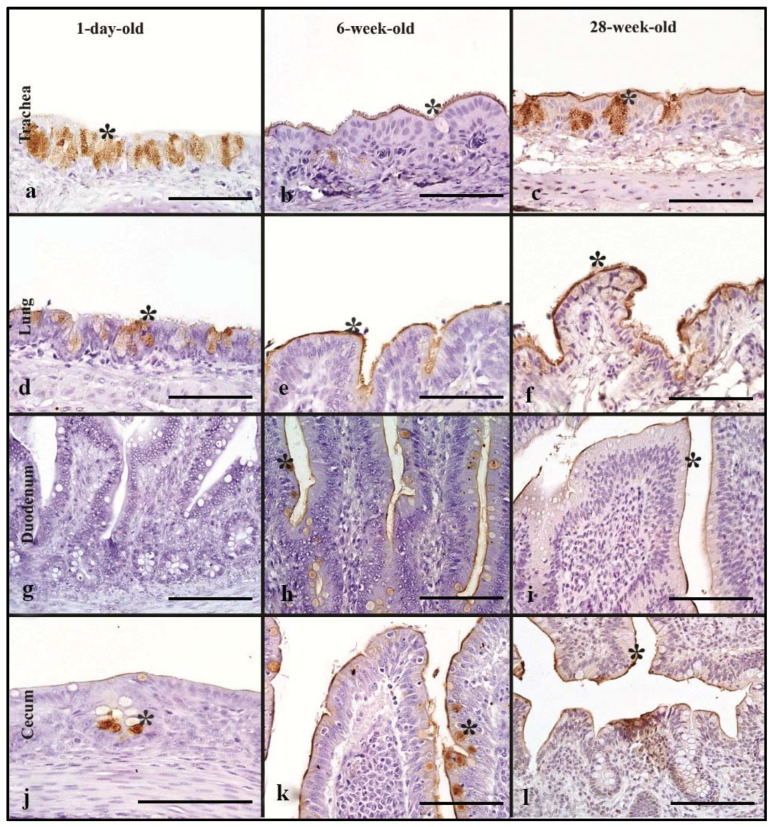
HPAIV H5 rHA binding in chickens of different age groups, denoted by dark brown staining using DAB- HRP via protein histochemistry denoted by asterisk (*). In the trachea, note strong rHA binding in 28-week-old layer chicken (**c**). In the lung, also note strong rHA binding to epithelial cells in 6-week-old and 28-week-old chickens (**e**,**f**). Significant rHA binding to the enterocytes of the duodenum in 6-week-old birds (**h**). The 6-week-old pullets have strong rHA binding to enterocytes and goblet cells of the cecum (**k**). Scale bar is 50 µm.

**Figure 5:**
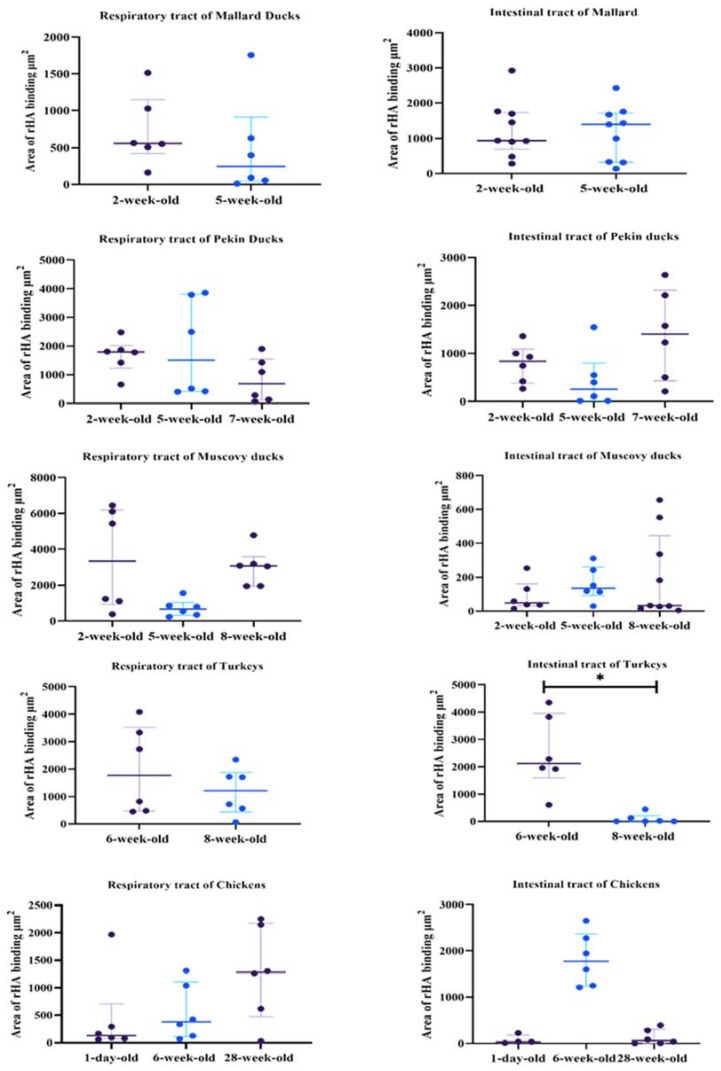
Dot plot charts showing the binding scores for HPAIV rHA in the respiratory (trachea and lung) and intestinal tract (upper intestine and lower intestine) of chickens, turkeys, and Mallard, Pekin, and Muscovy ducks. Dots represent the binding score for each tissue. Note significant age-related differences in MAB scores in the respiratory and intestinal tracts of the turkey. Statistical significance denoted by asterisks. Kruskal–Wallis test and Mann–Whitney U test (*p* ≤ 0.05) were used. Graphs formulated in Graph Pad Prism 9.0.0.(GraphPad Software, San Diego, CA, USA).

Protein histochemistry was used detect median area of binding (MAB) to respiratory (trachea, lung), intestinal (duodenum/jejunum, ileum/cecum) tract, and cloacal bursa. For comparison of age-related differences, chickens, commercial turkeys, Mallards, Pekin ducks, and Muscovy ducks (*Cairina moschata*) were used. Findings are presented in Table 1 and Table 2 and Figure 1, Figure 2, Figure 3, Figure 4 and Figure 5.

### 3.2. Mallards

Two-week-old and 5-week-old birds were used to determine age-related differences in MAB in Mallards. In the respiratory tract, rHA bound to the cilia and cell membrane of respiratory epithelial cells and the cytoplasm of goblet cells (Figure 1a,b). The 2-week-old and 5-week-old birds had moderate MAB scores in the trachea with no statistically significant differences between the age groups (Table 1). The lungs of the 2-week-old birds had strong MAB scores compared to 5-week-olds (Mann–Whitney test, *p* = 0.05). Next, the overall, cumulative MAB in the respiratory tract was determined by combining the MAB values for the trachea and lung. There was no statistically significant difference when the overall respiratory MAB was compared (Figure 5).

In the intestinal tract, the rHA bound to the surface cell membrane of enterocytes and the cytoplasm of goblet cells (Figure 1e–h). The MAB score was combined for the duodenum and jejunum (referred to as upper intestine) and the ileum and cecum (referred to as lower intestine). The 2-week-old birds had a significantly higher MAB score to the upper intestine compared to the lower scores seen in 5-week-old birds (Mann–Whitney test, *p* = 0.05, Table 1). However, in the lower intestine, both age groups had similar strong scores. There was no statistically significant difference in the combined intestinal MAB between the age groups (Figure 5). In the cloacal bursa, rHA binding was present on bursal epithelial cells. The 5-week-old mallards had significantly higher MAB scores compared to the 2-week-old birds (Mann–Whitney test, *p* = 0.05).

### 3.3. Pekin Ducks

Tissues from 2, 5, and 7-week-old Pekin ducks were used to determine age-related differences in rHA binding among age groups. The rHA bound to cilia and cell membrane of ciliated epithelial cells of the trachea, as well as to epithelial cells of the bronchi and parabronchi. Small amounts of rHA bound to the cytoplasm of goblet cells. Significant differences in the tracheal MAB scores were found between the 2-week-old birds compared to the 7-week-old group, with the 2-week-old Pekin ducks having higher scores (Kruskal–Wallis, *p* = 0.03), Dunn’s post hoc test, (Table 1). All age groups had strong to very strong MAB scores in the lung and no significant differences were found among the age groups (Figure 5). Furthermore, no significant differences in the cumulative respiratory MAB scores were observed among all age groups.

In the upper and lower intestine, rHA bound to the surface of enterocytes and the cytoplasm of goblet cells. In the upper intestine, there was no rHA binding in the 2-week-old or 5-week-old birds; however the 7-week-old birds had mild rHA binding, which was statistically significant (Kruskal–Wallis, *p* = 0.04, Dunn’s post hoc test). In the lower intestine, rHA binding ranged from mild to strong; however, there were no statistically significant differences between age groups (Figure 5). Concurrently, there were no statistically significant differences in the cumulative intestinal MAB scores among age groups. In the cloacal bursa, the 7-week-old birds also had the highest MAB scores; however, there were no statistically significant differences among age groups.

### 3.4. Muscovy Ducks

Birds from age groups 2-, 5-, and 8-weeks-old were used to evaluate rHA binding. In the respiratory tract, rHA was bound to the cilia and membrane of epithelial cells and the cytoplasm of goblet cells in the trachea, and lung (Figure 2a–c). In the trachea, a statistically significant difference in MAB scores was seen (Kruskal–Wallis test, *p* = 0.04) and with Dunn’s post hoc test, the 8-week-old Muscovy ducks (Figure 2f) had much higher scores when compared to the 5-week-old birds (Figure 2e, Table 1). In the lung, there was a significant difference in MAB scores (Kruskal–Wallis test *p* = 0.03). Using Dunn’s post hoc test, the 2-week-old ducks had significantly higher MAB scores than the 5-week-old birds. Evaluation of the cumulative respiratory MAB scores showed no statistically significant differences in the cumulative respiratory MAB scores.

In the intestine, the rHA bound to the surface of enterocytes and the cytoplasm of goblet cells, and to crypt epithelial cells (Figure 2g–l). The upper intestine had no statistically significant differences among age groups and there were mild MAB scores in all birds (Table 1). The lower intestine had a statistically significant difference among age groups (Kruskal–Wallis, *p* = 0.03), with 8-week-old birds having significantly higher MAB scores compared to 2-week-old birds. Evaluation of the cumulative MAB scores in the intestinal tract revealed no statistically significant difference among age groups. In the cloacal bursa, rHA binding to epithelial cells ranged from mild to very strong; however, there was no statistically significant difference among age groups (Figure 5).

### 3.5. Turkeys

Tissues from 6-week-old and 8-week-old turkeys were evaluated. The rHA bound to the ciliated epithelial cells, and on the membrane and in the cytoplasm of goblet cells in the respiratory tract (Figure 3). The trachea of the 8-week-old turkeys had significantly higher MAB scores compared to the 6-week-olds (Mann–Whitney test, *p* ≤ 0.05, Table 2). The 6-week-old birds had significantly higher MAB in the lung when compared to the 8-week-olds (Mann–Whitney test, *p* = 0.05). However, no significant difference was found when the cumulative respiratory tract MAB was compared between the age groups (Mann–Whitney test, *p* ≤ 0.05, Figure 5). 

The rHA binding in the intestinal tract localized to the membrane of enterocytes and to the cytoplasm of goblet cells (Figure 4e,f). The 6-week-olds had low MAB scores; however, 8-week-old duodenum could not be evaluated. In the lower intestine, the 6-week-old turkeys had significantly higher MAB scores compared to 8-week-olds (Mann–Whitney test, *p* = 0.01, Table 2). There was a statistically significant difference between the age groups when the cumulative intestinal MAB, with 6-week-old turkeys, having higher MAB compared to the 8-week-old birds (Mann–Whitney test, *p* ≤ 0.05, Figure 5). 

### 3.6. Chickens

For evaluation of differences in rHA binding in pullets and layers, 1-day-old, 6-week, and 28-week -old chickens were used. In the respiratory tract, rHA bound to the surface of ciliated epithelial cells as well as to cell membrane and cytoplasm of goblet cells and mucous glands (Figure 4a–c). In the trachea, the 28-week-old birds, had the highest MAB scores with no statistically significant difference among age groups (Table 2). Similarly, in the lung, the 28-week-old chicken, had the highest MAB scores, with no statistically significant difference. Additionally, the cumulative respiratory tissue MAB was not statistically difference among the age groups (Figure 5).

The HPAIV rHA bound to the cell membrane of enterocytes and the cytoplasm of goblet cells in the intestine of chickens (Figure 4h–l). In the upper intestinal tract, the 6-week-old birds had significantly higher MAB scores compared to the 1-day-old birds (Kruskal–Wallis, *p* = 0.03, Dunn’s post hoc test, Table 2). Likewise, the 6-week-old chickens had the highest MAB score in the lower intestine, which was significantly higher than the 28-week-old layers (Kruskal–Wallis, *p* = 0.05 Dunn’s post hoc test). The cumulative intestinal tract MAB scores were not statistically different among the age groups (Figure 5). In the cloacal bursa, the 1-day-old birds had higher MAB score compared to 6-week-old birds; however, this difference was not statistically significant.

### 3.7. Low Pathogenic Avian Influenza H5 rHA

**Figure 6:**
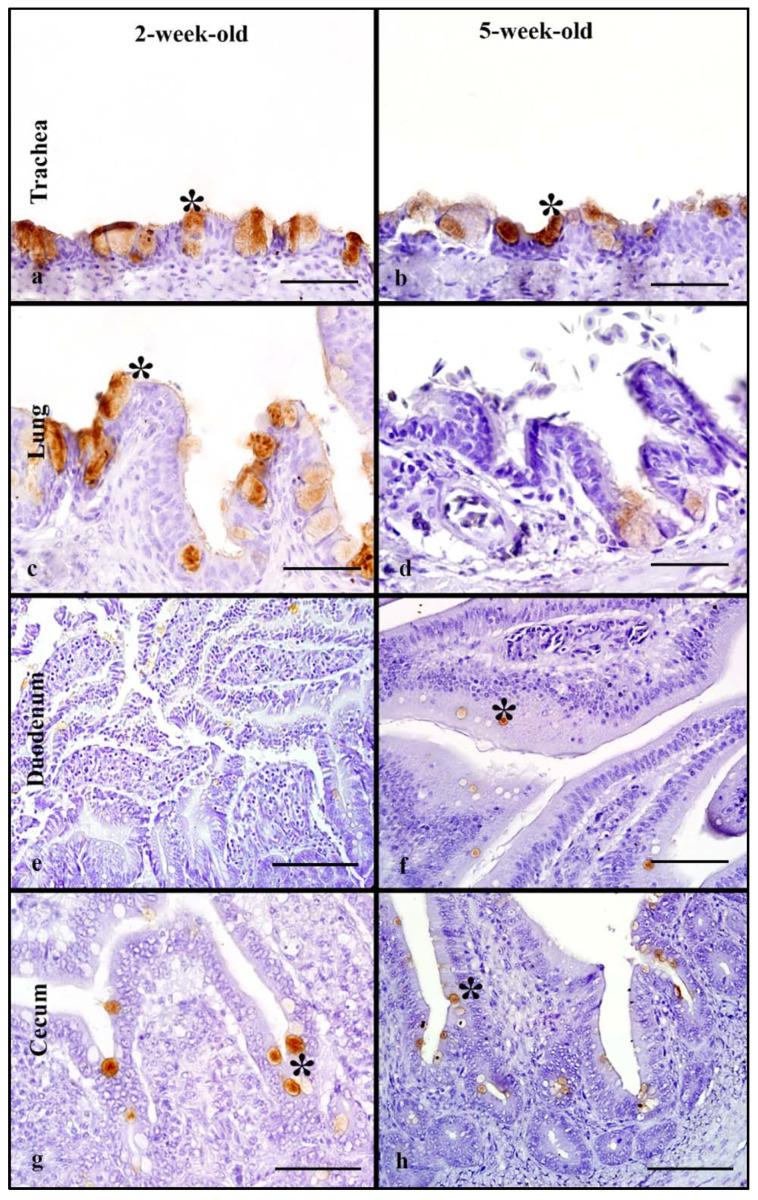
Surface area of LPAIV H5 rHA binding in Mallard ducks of different age groups, denoted by dark brown staining using 3,3-diaminobenzimidine (DAB) HRP via protein histochemistry, denoted by asterisk (*). Note mild to moderate rHA binding in the trachea and lung of 2-week-old and 5-week-olds mallards (**a**–**d**). In the cecum, similar mild rHA bindings is evident in 2- and 5-week-old birds (**e**). Scale bar is 50 µm.

**Figure 7:**
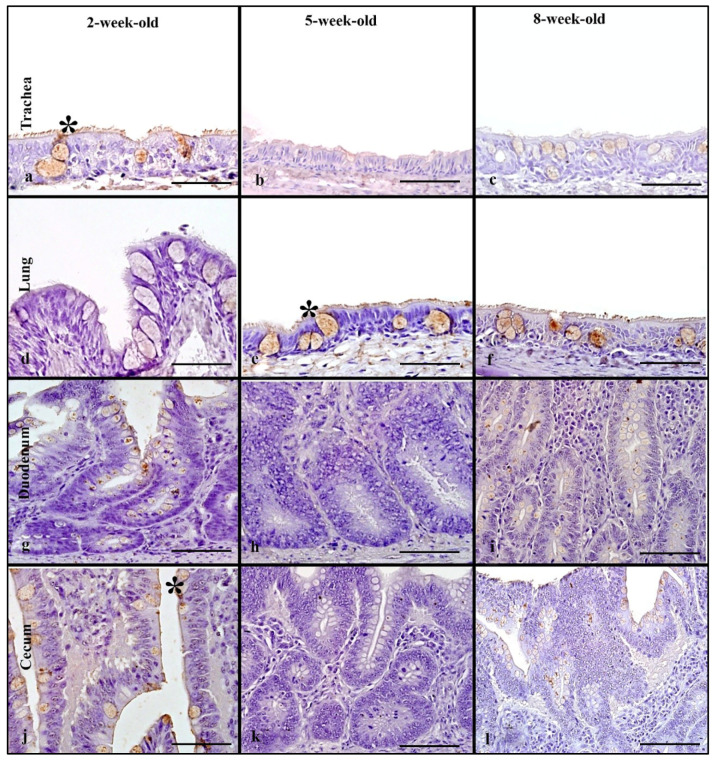
Surface area of LPAIV H5 rHA binding in Muscovy ducks of different age groups, visible as dark brown staining using 3,3-diaminobenzimidine (DAB) HRP via protein histochemistry, denoted by asterisk (*). Note mild levels of binding in the trachea of 5-week-old and 8-week-old Muscovy ducks, compared to moderate binding in the 2-week-old (**b**,**c**). Moderate levels of rHA binding is also visible in the lung of 5-week-old Muscovy duck (**e**). The level of rHA binding in the intestinal tract is generally mild (**g**–**l**). Scale bar is 50 µm.

**Figure 8:**
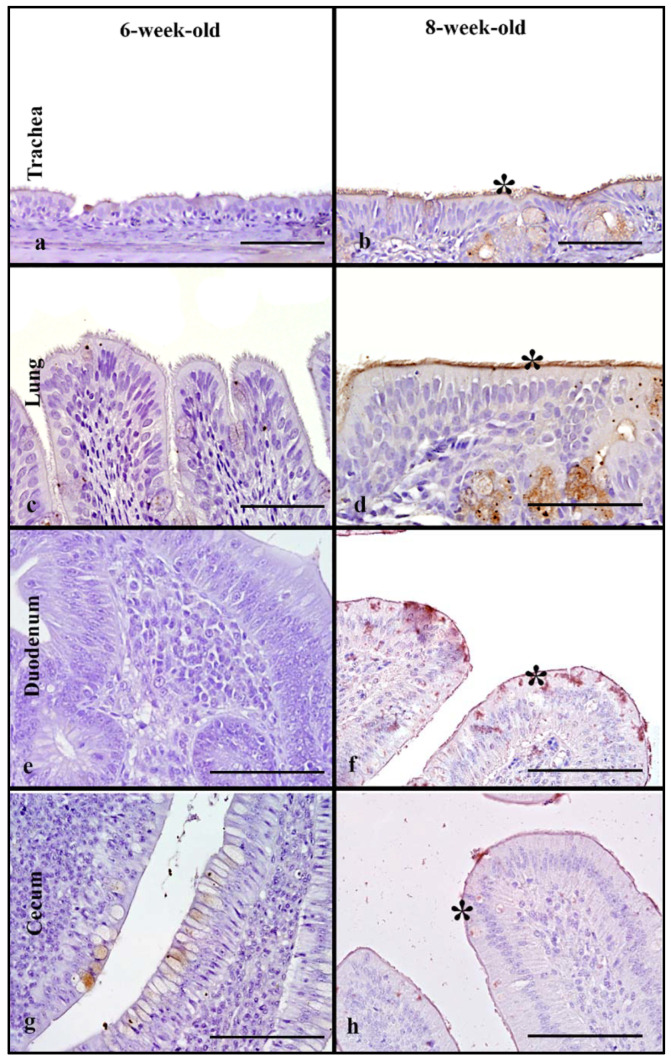
Surface area of LPAIV H5 rHA binding in Turkeys of different age groups, denoted by dark brown staining using DAB- HRP via protein histochemistry denoted by asterisk (*). Note moderate and very strong levels of rHA binding in the trachea and lung of 8-week-old and 6- week-old birds to respiratory epithelial cells (**b**,**d**). In the lower intestine, rHA binding is mild in both age groups (**e**–**h**). Scale bar is 50 µm.

**Figure 9:**
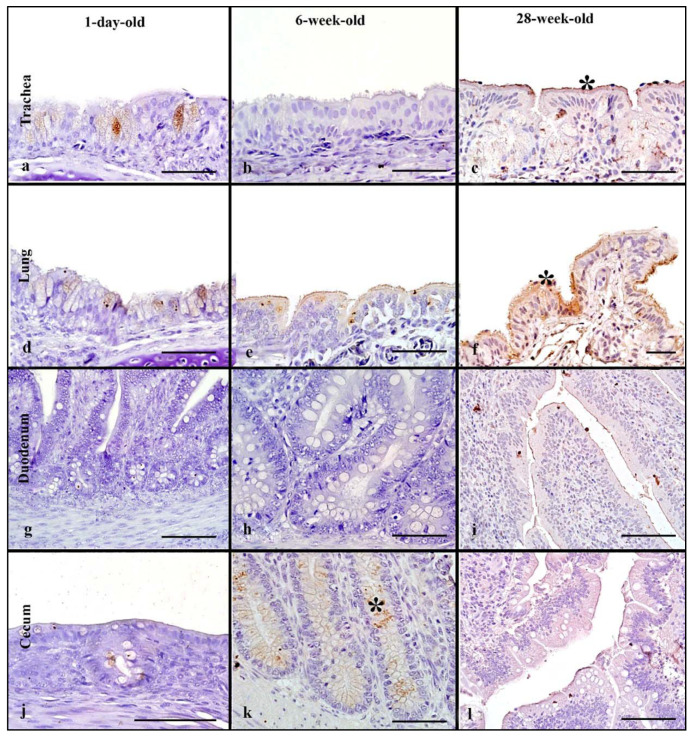
Surface area of LPAIV H5 rHA binding in chickens of different age groups, visible as dark brown staining using DAB-HRP via protein histochemistry, denoted by asterisk (*). Note generally mild levels of rHA binding in all tissues of the age groups to the respiratory epithelial cells (**a**,**c**–**e**), enterocytes (**i**) and goblet cells (**j**,**k**). Scale bar is 50 µm.

**Figure 10:**
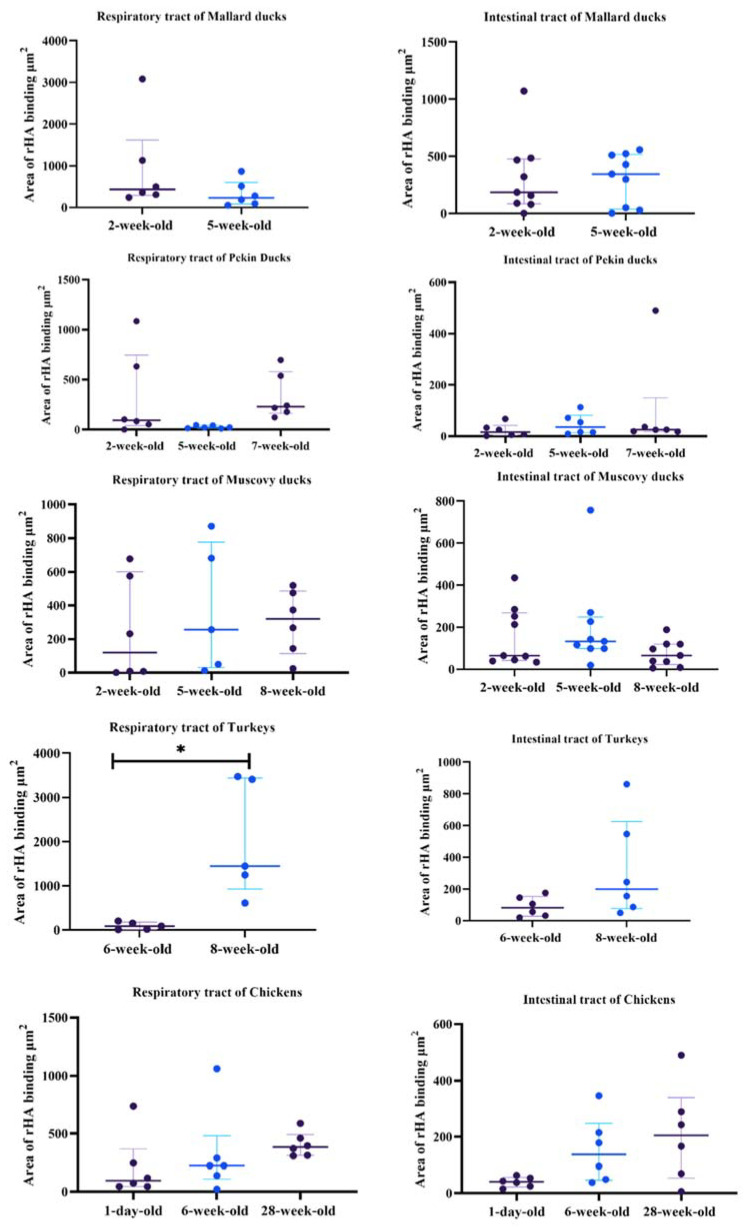
Dot plot charts show the binding scores of LPAIV rHA binding to the respiratory (trachea and lung) and intestinal tract (upper and lower intestine) of Mallards, Muscovy ducks, Pekin ducks, chickens, and turkeys. Dots represent the binding score for each tissue. A significant difference is seen only in the respiratory tract of turkeys. Statistical significance denoted by asterisk and bars. Kruskal–Wallis test and Mann–Whitney U test (*p* ≤ 0.05). Graphs formulated in Graph Pad Prism 9.0.0. (GraphPad Software, San Diego, CA, USA).

Protein histochemistry was used detect median area of binding (MAB) to respiratory (trachea, lung), intestinal (duodenum/jejunum, ileum/cecum) tract, and cloacal bursa. For comparison of age-related differences, chickens, commercial turkeys, Mallards, Pekin ducks, and Muscovy ducks were used. These findings are presented in Table 3 and Table 4 and Figure 6, Figure 7, Figure 8, Figure 9 and Figure 10.

### 3.8. Mallards

Tissues from 2- and 5-week-old mallards were used to evaluate differences in rHA binding among age groups. In the respiratory tract, the LPAIV rHA bound to the membrane of epithelial cells and to the cytoplasm of goblet cells in the trachea, and secondary and tertiary bronchi of the lung (Figure 6a–d). The trachea of both age groups had mild MAB, and no significant differences were seen (Table 3). In the lung, 2-week-old mallards had higher MAB compared to 5-week-olds with no statistically significant difference. Moreover, the cumulative respiratory MAB had no significant differences between age groups (Figure 10).

In the intestinal tract, the LPAIV rHA bound to the surface of enterocytes and to the cytoplasm of goblet cells (Figure 6e–h). Both age groups had mild MAB in the upper intestine; however, the 2-week-olds had significantly higher MAB compared to 5-week-olds (Mann–Whitney test, *p* ≤ 0.05, Table 3). In the lower intestine, the MAB was also mild in both age groups and there were no statistically significant differences. The cumulative intestinal MAB had no statistically significant difference between the age groups (Figure 10). In both age groups, the cloacal rHA binding to epithelial cells was mild with no statistically significant difference. 

### 3.9. Pekin Ducks

Tissue from 2-, 5-, and 7-week-old birds were used for evaluation of LPAIV rHA binding. In the respiratory tract of Pekin ducks, LPAIV rHA binding was mostly present on the cytoplasm of goblet cells and the epithelium of parabronchi. No significant differences were seen in the tracheal MAB among the age groups (Table 3). Similarly, there were no significant differences in rHA binding in the lung among age groups and all birds had mild rHA binding. When the cumulative rHA binding in the respiratory tract was compared, no statistically significant difference was found (Figure 10).

Intestinal LPAIV rHA binding in Pekin ducks was present on the surface of enterocytes and the cytoplasm of goblet cells. In the upper intestine, generally, MAB scores were mild, and there was no significant difference among the age groups. In the lower intestine, the MAB was also mild across age groups, and no significant differences were seen. Comparison of the cumulative intestinal MAB among the age groups revealed no significant differences. Additionally, the cloacal bursa had no significant differences in the MAB across the age groups.

### 3.10. Muscovy Ducks

Tissues from 2, 5, and 7-week-old birds were used for evaluation of LPAIV rHA MAB among age groups. Binding was observed predominantly in the cytoplasm of goblet cells, and on the surface of ciliated epithelial cells (Figure 7a–f). In the trachea, there was mild to moderate LPAIV rHA binding among all age groups; however, MAB in 2-week-old Muscovy ducks was significantly higher than in 5-week-old ducks (Kruskal–Wallis, *p* = 0.001, Dunn’s post hoc test, Table 3). In the lung, there were no significant differences among the age groups. No significant difference in the cumulative respiratory MAB was found among the groups.

In the intestine, LPAIV rHA binding was present on the surface of enterocytes and the cytoplasm of goblet cells (Figure 7g–l). In the upper intestine, all birds had mild MAB scores and no significant differences among the age groups (Table 3). Similarly, mild MAB scores were seen in the lower intestine across all age groups with statistically significant differences. Comparison of the cumulative intestinal MAB scores revealed no significant differences among age groups. The cloacal bursa had mild MAB scores with no significant differences among the age groups (Figure 10).

### 3.11. Turkeys

LPAIV rHA binding in turkeys was evaluated using 6-week-old and 8-week-old turkeys. rHA binding to the respiratory tract of turkeys was localized to the ciliated epithelial cells, goblet cells, and mucous glands of the trachea and respiratory epithelium of secondary and tertiary bronchi (Figure 8a–d). In tracheas and lungs, significantly higher MAB scores were observed in 8-week-old birds compared to 6-week-olds (Mann–Whitney test, *p* = 0.05, Table 4). A significant difference in the cumulative respiratory tract MAB scores between the 8-week-old and 6-week-old turkeys was seen (Mann–Whitney test, *p* = 0.05), with higher cumulative respiratory MAB in the 8-week-olds (Figure 10).

In the intestinal tract, rHA bound to enterocytes, and goblet cells. In the upper intestine, (Figure 8e–h) the rHA binding was mild, and no significant differences was noted between age groups. In the lower intestine, no significant differences were seen and rHA binding ranged from mild to moderate (Table 4). There were no statistically significant differences in the cumulative intestinal tract rHA among the age groups. In the cloacal bursa, both 6-week-old and 8-week-old birds had mild MAB scores, with rHA localized to bursal epithelial cells; however, due to small sample size for bursas of 6-week-olds, statistical analyses were not performed. 

### 3.12. Chickens

One-day-old, 6-week-old, and 28-week-old chickens were used to evaluate LPAIV rHA binding across age groups. The rHA binding to the respiratory tract of chickens was mild and localized to the ciliated epithelial cells, goblet cells, and the respiratory epithelium of secondary and tertiary bronchi (Figure 9a–f). In the trachea, a significant difference was evident between 1-day-old and 28-week-old birds, with 28-week-old chickens having higher MAB (Kruskal–Wallis, *p ≤* 0.04, Table 4). In the lung, no major differences were observed in the MAB across age groups. When the respiratory tissues were combined, no statistically significant difference was seen (Figure 10).

In the intestine, LPAIV rHA bound to the cytoplasm of goblet cells and the surface of enterocytes (Figure 9g–l). All age groups had mild MAB and no significant differences were seen among age groups in both upper and lower intestines (Table 4). However, evaluation of cumulative intestinal MAB revealed no statistically significant difference among the age groups (Figure 10). 

The cloacal bursa of 1-day old and 6-week-old birds had mild MAB scores and binding was present mainly on bursal epithelial cells and goblet cells. There were no statistically significant differences in cloacal bursa scores among age groups.

## 4. Discussion

We demonstrate age-related differences in rHA tissue binding across respiratory, intestinal, and cloacal bursal tissues in some *Anseriformes* and gallinaceous poultry species. Using the HPAIV H5 rHA, the younger age group of turkeys (6 weeks old) had higher levels of binding in the lung. However, the opposite was seen with the LPAIV rHA, where the older age group had higher levels of binding to the respiratory tract. This can suggest that the HA binding is not only related to host factors such as sialic acid distribution and glycosylation of the host tissues but also to the receptor binding site of the particular HA and the host susceptibility. This is also supported by Kimble et al. where the distribution of sialic acid receptors in turkeys was not found to be age dependent [11]. Outbreaks of both LPAIV and HPAIV are common in turkey flocks [35]. Spackman et al. characterized the pathobiology of clade 2.3.4.4 H5N2 viruses in 4-week-old broad breasted white turkeys using A/northern pintail/WA/40964/2014 (duck isolate used in this study), A/chicken/IA/13388/2015 and A/turkey/MN/12528/2015 [35]. While high levels of antigen were detected via immunohistochemistry in the brain, heart, pancreas, and adrenal glands, varying levels of antigen were seen in the trachea, lung, intestine, and cloacal bursa. Another interesting finding in the former study is the high titers of virus shed via the cloacal route, which is less common in gallinaceous birds infected with HPAIV, but is in agreement with the finding in this present study of high MAB scores in the cecum of 6-week-old birds. In a recent study, 6-week-old turkeys had shorter mean death times, and succumbed to infection with clade 2.3.4.4 A HPAIV H5N2 (A/turkey/Minnesota/12582/2015), compared to 16-week-old birds. The later had more histologic lesions of severe inflammation, hemorrhage, and necrosis in the heart, spleen, pancreas, and liver, which is in agreement with the findings of the present study [24]. Histopathology and immunohistochemistry (IHC) for detection of AIV antigen showed strong viral staining in the lung, which is in agreement with high levels of recombinant HA binding to bronchial epithelial cells demonstrated in the present study. The cecum of infected turkeys, however, had moderate IHC scores, in contrast to the strong rHA binding as seen in our study. This could be due to the virus used in the previously mentioned study being isolated from turkeys, and therefore differences in the pathobiology and viral antigen distribution could have occurred due to viral adaptation to the host or the lack of virus spread in the gastrointestinal tract. Moreover, very strong rHA binding was detected in the trachea of 6-week-old turkeys of our study, which was not observed in experimentally infected turkeys [24]. In another study, Santos et al. found that 16-week-old turkeys had higher virus titers in the trachea compared to a 6-week-old group of birds at 2 days post infection with clade 2.3.4.4 H5N2 virus. Moreover, the younger group of birds succumbed to infection more rapidly compared to the older age group [36]. Higher viral titers in the trachea of younger birds agrees with our study, seen with 6-week-old birds having significantly higher MAB scores compared to the 8-week-old birds. 

In contrast to our finding and previous studies using H5N2 viruses, age-related differences in susceptibility of commercial turkeys to infection during an outbreak of H7N7 HPAIV in the Netherlands have been described by Elbers et al. However, in this outbreak, birds greater than 16 weeks of age were more severely affected compared to the birds less than 11 weeks of age. This suggests that age-related differences in susceptibility to infection can also be dependent on the particular HA subtype [37]. With respect to sialic acid distribution, Kimble et al. examined the sialic acid (SA) receptors in turkeys and found both α2, 3 and α2, 6-linked sialic acid receptors in the respiratory and intestinal tract [11]. Particularly, the trachea had moderate to high levels of expression of α2, 3 sialic acid receptors which agrees with the HA binding patterns seen in our study. However, in the present study, 6-week-old turkeys had markedly higher levels of HPAIV rHA binding to the intestinal tract, especially to the lower intestine. This is not completely explained by the presence of the SA receptor, as an age dependent increase in α2, 3-linked SA was not described in the previously mentioned study. These differences can explain why the binding results are different for the HPAIV rHA and LPAIV rHA using the same tissues from the same birds.

In mallard and Muscovy ducks, higher levels of HPAIV rHA bound to the respiratory tract of the youngest (2-week-old) age group of birds. Experimental infection of 2-week-old mallard ducks with clade 2.3.4.4 H5N2 virus showed that infected birds did not develop fever and shed viruses for prolonged periods via the oropharyngeal and cloacal routes. The shed viruses also efficiently transmitted to contact 2-week-old mallards, which supports a high susceptibility of this age group of infection [22]. França et al. demonstrated that 4-week-old mallards had strong expression of α2, 3-linked SA in the respiratory tract and intestinal tract, which correlates with the strong levels of rHA binding in these tissues [10]. Additionally, França et al. quantified α2, 3-linked SA receptors present in respiratory tract, intestinal tract and bursa of Fabricius of 2-, 4-, 8-, 12-, and 16-week-old mallard ducks [15]. In this study, similar strong levels of α2, 3-linked SA receptors were present across the age groups in the respiratory tract, in ciliated epithelial cells, goblet cells, and the mucus glands. A change in lectin binding was determined in the nasal turbinate from the 2-week-old birds compared to the older birds. In the small intestine, strong level of α2, 3-linked SA receptors were seen in goblet cells. Additionally, there was strong expression of α2, 3-linked SA receptors, in enterocytes and goblet cells of the ileum, cecum, and colon. Strong levels of α2, 3-linked SA receptors were also reported in bursal epithelial cells in the mallard ducks of this study. Muscovy ducks are highly susceptible to infection with HPAIV and may have more severe clinical disease compared to Mallard and Pekin duck [38,39,40,41]. These results suggest that young Muscovy ducks may be even more susceptible compared to young mallards.

In Pekin ducks, there were no overall differences in HPAIV rHA binding to the respiratory and intestinal tracts across different age groups. This is in partial agreement with the age-related differences in expression of sialic acid receptors described by Kimble et al., where there was no difference in the expression of α2, 3 or α2, 6-linked SA in the trachea [11]. However, in the former study, found an age dependent increase in α2, 3-linked SA in the lung and large intestine, and 2- and 4-week-old Pekin ducks, had higher α2, 3-linked SA levels compared to day-old ducks. In addition, in using Pekin ducks, Pantin-Jackwood et al. demonstrated that 2-week-old Pekin ducks were more susceptible to infection and succumbed earlier to clinical disease; however, this finding was attributed to the immature immune system of the younger ducks compared to the older counterparts [20,42].

Six-week-old chickens had significantly higher levels of HPAIV rHA binding to the intestinal tract compared to other age groups. Evaluation of the sialic acid receptor changes with age, demonstrated that 4-week-old chickens had a slightly lower percentage of avian receptor in the bronchi, small intestine and large intestine, which further decreased in the bronchi, and small intestine of adult layers [26]. In contrast, a previous study demonstrated that there was an increase in the α2, 6-linked SA in the large intestine of 4-week-old chickens compared to 1-day old chickens, which dropped to very low levels adult chicken layers [26]. Since the SA receptor distribution in 6-week-old chickens was not studied, it may not be accurate to extrapolate data from 4-week-old birds. However, experimental infection of 6-week-old SPF-layer chickens, with clade 2.3.4.4 H5 viruses, have shown high titers of virus shed via both the oropharyngeal and cloacal route and efficient transmission to contact chickens, which is in agreement with the intestinal tropism of the HPAIV rHA detected in our study [43]. We found no overall significant difference in the respiratory and intestinal rHA binding, which is supportive of the findings of no age-associated susceptibility of broiler chickens to clade 2.3.4.4A. However, we have demonstrated in a previous study, that differences in rHA binding could be seen between broiler and egg-laying chicken breeds [32]. 

Using the LPAIV H5 rHA, there were individual variations among each species. For instance, mallards had no significant age-related differences in LPAIV H5 rHA binding. This finding is in agreement with a previous study by Costa et al. using several H5 LPAIV viruses, where age did not affect the ability of mallards to become infected with H5N2 LPAIV. The 4-week-old birds in the study, however, consistently shed virus via the cloacal route [30]. In the wild, young mallards are more susceptible to infection; however, this is largely due to the immaturity of the host immune system and the lack of prior exposure to AIV as opposed to viral/HA binding, compared to adult ducks, which most likely have been exposed to one or more avian influenza viruses [19]. 

One drawback of this study is the relatively small sample size of birds examined for each age group that could impair extrapolation to all birds within the species; however, this study serves as the foundation for future studies, where larger numbers of birds per species could be examined. Another drawback is that we only used one representative HPAIV and LPAIV for the comparisons, ideally, the low pathogenic and highly pathogenic version of the same virus could be used in future studies. It is known that influenza viruses can become host adapted which could affect the HA binding, therefore viruses from different origins such chicken, duck, and turkey can be used for future studies.

It is important to note that factors other than virus receptor binding affect host susceptibility of live birds to infection and clinical disease. Route of exposure, host adaptability of the virus, the action of host immunity, mutations in the receptor binding site, and glycosylation of the HA and NA should all be considered in determining tissue tropism and the susceptibility of birds to AIV infections. 

## 5. Conclusions

This study supports an age-related susceptibility of turkeys to HPAIV, which has been previously described for clade 2.3.4.4. viruses. Younger turkeys had higher respiratory and intestinal HPAI rHA binding compared to the older birds. In contrast, older turkeys had higher overall respiratory LPAIV rHA binding compared to younger birds. Among the species evaluated, some age-related variations in recombinant hemagglutinin tissue binding were detected in domestic poultry and waterfowl species; however, statistically significant differences were not always present. These findings reinforce previous experimental trials and field outbreaks with some exceptions.

## Figures and Tables

**Table 1:** Median area of binding (MAB) in μm^2^ of HPAIV rHA binding in the trachea, lung, small intestine, large intestine, and cloacal bursa of Mallards, Pekin ducks, and Muscovy ducks of different age groups.

Median Area of HPAIV rHA Binding in μm^2^								
Tissue	Mallard		Pekin Duck			Muscovy Duck		
	2-Week-Old (*n* = 3)	5-Week-Old (*n* = 3)	2-Week-Old (*n* = 3)	5-Week-Old (*n* = 3)	7-Week-Old (*n* = 3)	2-Week-Old (*n* = 3)	5-Week-Old (*n* = 3)	8-Week-Old (*n* = 3)
Trachea	509	631	1421 *	420	133 *	1117	349 *	1953 *
Lung	1028 ^†^	92 ^†^	1805	3789	1423	6105 *	860 *	3184
Duodenum/ Jejunum	927 ^†^	316 ^†^	0 *	0 *	16 *	130	119	30
Ileum/ Cecum	1569	1546	836	252	1402	37 *	242	444 *
Cloacal Bursa	146 ^†^	1581 ^†^	164	227	273	421	1692	380

Scale: 0–10 μm^2^: No binding; >10–500 μm^2^: Mild; >500–1000 μm^2^: Moderate; >1000–1500 μm^2^: Strong; >1500 μm^2^: Very Strong; * = Kruskal–Wallis, *p* ≤ 0.05, ^†^ = Mann–Whitney U test, *p* ≤ 0.05.

**Table 2:** Median area of binding (MAB) in μm^2^ of HPAIV rHA binding in the trachea, lung, small intestine, large intestine and cloacal bursa of turkeys and chickens of different age groups.

Median Area of HPAIV rHA Binding in μm^2^					
Tissue	Turkey		Chicken		
	6-Week-Old (*n* = 3)	8-Week-Old (*n* = 3)	1-Day-Old (*n* = 3)	6-Week-Old (*n* = 3)	28-Week-Old (*n* = 3)
Trachea	477 ^†^	1720 ^†^	287	121	1259
Lung	3332 ^†^	566 ^†^	74	1038	1302
Duodenum/ Jejunum	49	ND	34 *	1251*	313
Ileum/ Cecum	2121 ^†^	71 ^†^	149	2654 *	7 *
Cloacal Bursa	ND	135	6347	10	ND

Scale: 0–10 μm^2^: No binding; >10–500 μm^2^: Mild; >500–1000 μm^2^: Moderate; >1000–1500 μm^2^: Strong; >1500 μm^2^: Very Strong; * = Kruskal–Wallis, *p* ≤ 0.05, ^†^ = Mann–Whitney U test, *p* ≤ 0.05.

**Table 3:** Median area of binding (MAB) in μm^2^ of LPAIV rHA in the trachea, lung, small intestine, large intestine, and cloacal bursa of Mallard ducks, Pekin ducks, and Muscovy ducks of different age groups.

Median Area of LPAIV rHA binding in μm^2^								
Tissue	Mallard		Pekin Duck			Muscovy Duck		
	2-Week-Old (*n* = 3)	5-Week-Old (*n* = 3)	2-Week-Old (*n* = 3)	5-Week-Old (*n* = 3)	7-Week-Old (*n* = 3)	2-Week-Old (*n* = 3)	5-Week-Old (*n* = 3)	8-Week-Old (*n* = 3)
Trachea	360	92	632	21	539	578 *	31 *	143
Lung	719	277	84	22	178	7	681	474
Duodenum/ Jejunum	90 ^†^	29 ^†^	7	15	19	65	99	120
Ileum/ Cecum	394	469	25	71	36	157	138	96
Cloacal Bursa	20	28	164	36	90	0	404	149

Scale: 0–10 μm^2^: No binding; >10–500 μm^2^: Mild; >500–1000 μm^2^: Moderate; >1000–1500 μm^2^: Strong; >1500 μm^2^: Very Strong; * = Kruskal–Wallis, *p* ≤ 0.05, ^†^ = Mann–Whitney U test, *p* ≤ 0.05.

**Table 4:** Median area of binding (MAB) in μm^2^ of LPAIV rHA binding in the trachea, lung, small intestine, large intestine, and cloacal bursa of turkeys and chickens of different age groups.

Median Area of LPAIV rHA binding in μm^2^					
Tissue	Turkey		Chicken		
	6-Week-Old (*n* = 3)	8-Week-Old (*n* = 3)	1-Day-Old (*n* = 3)	6-Week-Old (*n* = 3)	28-Week-Old (*n* = 3)
Trachea	179 ^†^	928 ^†^	45 *	222	395 *
Lung	20 ^†^	3408 ^†^	246	139	313
Duodenum/ Jejunum	31	86	43	95	70
Ileum/ Cecum	145	546	25	179	289
Cloacal Bursa	72	457	33	107	N/A

Scale: 0–10 μm^2^: No binding; >10–500 μm^2^: Mild; >500–1000 μm^2^: Moderate; >1000–1500 μm^2^: Strong; >1500 μm^2^: Very Strong. N/A: not applicable; * = Kruskal–Wallis, *p* ≤ 0.05, ^†^ = Mann–Whitney U test, *p* ≤ 0.05.

## Data Availability

Data is contained within the article.
